# BCL-xL/BCL2L1 is a critical anti-apoptotic protein that promotes the survival of differentiating pancreatic cells from human pluripotent stem cells

**DOI:** 10.1038/s41419-020-2589-7

**Published:** 2020-05-18

**Authors:** Larry Sai Weng Loo, Andreas Alvin Purnomo Soetedjo, Hwee Hui Lau, Natasha Hui Jin Ng, Soumita Ghosh, Linh Nguyen, Vidhya Gomathi Krishnan, Hyungwon Choi, Xavier Roca, Shawn Hoon, Adrian Kee Keong Teo

**Affiliations:** 1 Stem Cells and Diabetes Laboratory, Institute of Molecular and Cell Biology, A*STAR, Proteos, Singapore, 138673 Singapore; 2 School of Biological Sciences, Nanyang Technological University, Singapore, 637551 Singapore; 3 Computational and Statistical Systems Biology, Institute of Molecular and Cell Biology, A*STAR, Proteos, Singapore, 138673 Singapore; 4 Department of Biochemistry, Yong Loo Lin School of Medicine, National University of Singapore, Singapore, 117596 Singapore; 5 Molecular Engineering Lab, Proteos, Singapore, 138673 Singapore; 6 Department of Medicine, Yong Loo Lin School of Medicine, National University of Singapore, Singapore, 119228 Singapore

**Keywords:** Differentiation, Stem-cell differentiation

## Abstract

The differentiation of human pluripotent stem cells into pancreatic cells involves cellular proliferation and apoptosis during cell fate transitions. However, their implications for establishing cellular identity are unclear. Here, we profiled the expression of BCL-2 family of proteins during pancreatic specification and observed an upregulation of BCL-xL, downregulation of BAK and corresponding downregulation of cleaved CASP3 representative of apoptosis. Experimental inhibition of BCL-xL reciprocally increased apoptosis and resulted in a decreased gene expression of pancreatic markers despite a compensatory increase in anti-apoptotic protein BCL-2. RNA-Seq analyses then revealed a downregulation of multiple metabolic genes upon inhibition of BCL-xL. Follow-up bioenergetics assays revealed broad downregulation of both glycolysis and oxidative phosphorylation when BCL-xL was inhibited. Early perturbation of BCL-xL during pancreatic specification also had subsequent detrimental effects on the formation of INS^+^ pancreatic beta-like cells. In conclusion, the more differentiated pancreatic progenitors are dependent on anti-apoptotic BCL-xL for survival, whereas the less differentiated pancreatic progenitors that survived after WEHI-539 treatment would exhibit a more immature phenotype. Therefore, modulation of the expression level of BCL-xL can potentially increase the survival and robustness of pancreatic progenitors that ultimately define human pancreatic beta cell mass and function.

## Introduction

Human pluripotent stem cells (hPSCs), an umbrella term for human embryonic stem cells (hESCs) and human-induced pluripotent stem cells (hiPSCs), are now routinely differentiated into desired human cell types for studying human organ development, in vitro disease modeling or even potential cell replacement therapy^1^. In the context of diabetes, hPSCs can now be differentiated into pancreatic progenitors with ease and subsequently to achieve 20–40% insulin^+^ pancreatic beta-like cells^2^.

During the exit from pluripotency toward lineage specification, a significant amount of cell death is typically observed^3,4^. While programmed cell death or apoptosis is well-known to be involved in developmental processes^5^, detailed mechanisms, especially in the context of hPSC differentiation, remain to be elucidated. In particular, a combination of cellular apoptosis, differentiation, and proliferation could be taking place concurrently as hPSCs are being directed into a particular lineage such as the pancreatic cells.

The BCL-2 family of proteins are notable for their involvement in the promotion or inhibition of apoptosis^6^. BAX and BAK are the two main multidomain pro-apoptotic members required for the execution phase of the mitochondrial apoptosis pathway^7^, whereas BCL2, BCL-xL (gene/transcript name *BCL2L1*), MCL1, BCL2A1, and BCL-W are the common anti-apoptotic proteins that promote cell survival^8^. The balance between the anti-apoptotic and pro-apoptotic members ultimately determines whether a cell lives or dies. For instance, BCL2 is known to promote the survival of hESCs^9^, whereas pro-apoptotic BAX is known to cull hESCs with genomic mutations and DNA damage rapidly^10^. Currently, the roles of these BCL-2 family of proteins in hPSC lineage differentiation are poorly understood.

Here, we differentiated hPSCs into pancreatic progenitors and uncovered a unique reciprocal relationship between anti-apoptotic BCL-xL and pro-apoptotic BAK proteins. BCL-xL was found to be important in the early pancreatic progenitor stage in suppressing cleaved caspase-3 activation. When BCL-xL expression or function was inhibited experimentally, early human pancreatic differentiation from hPSCs was disrupted, leading to metabolic alterations and less efficient formation of insulin^+^ pancreatic beta-like cells. Together, we report a previously underappreciated role of BCL-xL in promoting the survival of differentiating pancreatic progenitors from hPSCs. Well-differentiated pancreatic progenitors are highly dependent on BCL-xL for their pancreatic identity as determined by their pancreatic marker gene expression. Therefore, modulation of the expression level of BCL-xL during hPSC differentiation may be one potential means to improve the efficiency of differentiation and survival of human pancreatic progenitors.

## Results

### Anti-apoptotic BCL-xL and pro-apoptotic BAK proteins exhibit opposite trends during pancreatic specification from hPSCs

**Fig. 1:**
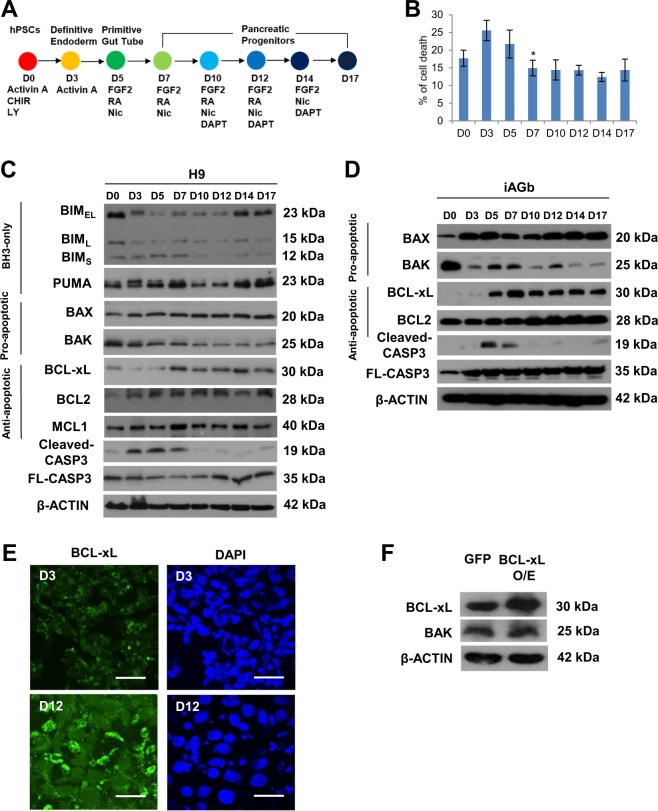
Anti-apoptotic BCL-xL and pro-apoptotic BAK proteins exhibit opposite trends during pancreatic specification from human pluripotent stem cells. **a** Schematic showing 17D differentiation protocol used to generate early pancreatic progenitors. Respective growth factors used are depicted at each time point. **b** Trypan blue staining showing the % of cell death over the course of 17D differentiation. Error bars indicate standard deviation of three biological replicates undergoing independent differentiations. Asterisk (*) indicates *P* < 0.05 compared between D5 and D7. Western blot showing the expression of BCL-2 proteins over the course of 17D differentiation in **c** H9 hESCs and **d** iAGb hiPSCs. **e** Immunofluorescence staining for BCL-xL protein on D3 and D12 cells. Scale bar represents 50 μm. **f** Western blot showing the expression of BAK protein upon overexpression of BCL-xL in undifferentiated hPSCs. “See also Fig. S1”.

During our routine differentiation of hPSCs into pancreatic progenitors using our previously described 17-day (D) differentiation protocol (Fig. 1a, S1a–c)^11,12^, we typically observe increased cell death in the first 5 days marking the transition from pluripotency at D0–D5 primitive gut tube before a significant decrease in cell death starting from D7 early pancreatic progenitors (Fig. 1b). To understand the molecular changes underlying this phenomenon, we decided to evaluate the protein expression profile of several common members of the BCL-2 family (Fig. 1c, d). After washing away the dead cells, the protein expression of the live cells was assessed using western blot analysis. The BH3-only proteins BIM and PUMA did not reflect reproducible changes from D0 to D17 of pancreatic differentiation. Although pro-apoptotic proteins BAX and BAK are known to heterodimerize to execute apoptosis, we found BAX to be expressed at constant levels whereas BAK was surprisingly decreased from D7 onwards. Conversely, multidomain anti-apoptotic protein BCL-xL exhibited a reciprocal increase in protein expression from D7 onwards, whereas anti-apoptotic protein BCL2 or MCL1 were unchanged throughout differentiation (Fig. 1c, d). These observations were confirmed in both H9 hESCs (Fig. 1c) and another independent hiPSC line, iAGb (Fig. 1d). This reciprocal upregulation of BCL-xL and downregulation of BAK protein expression coincided with a decrease in cleaved CASP3 expression from D7 onwards (Fig. 1c, d), suggesting the unique importance of BCL-xL but not BCL2 or MCL1 in promoting the survival of differentiating pancreatic progenitors from D7. Immunostaining analyses also confirmed an upregulation of BCL-xL protein from D3 to D12 (Fig. 1e). To confirm if there is a direct reciprocal relationship between BCL-xL and BAK, we overexpressed BCL-xL in undifferentiated hPSCs and evaluated the expression of BAK. While BCL-xL protein was successfully overexpressed, there was no appreciable effect on BAK protein expression (Fig. 1f), suggesting that this reciprocal relationship is indirect. Together, these data suggest that anti-apoptotic BCL-xL protein could be involved during early human pancreatic specification in culture.

### Experimental inhibition of BCL-xL protein function increases apoptosis during pancreatic specification

**Fig. 2:**
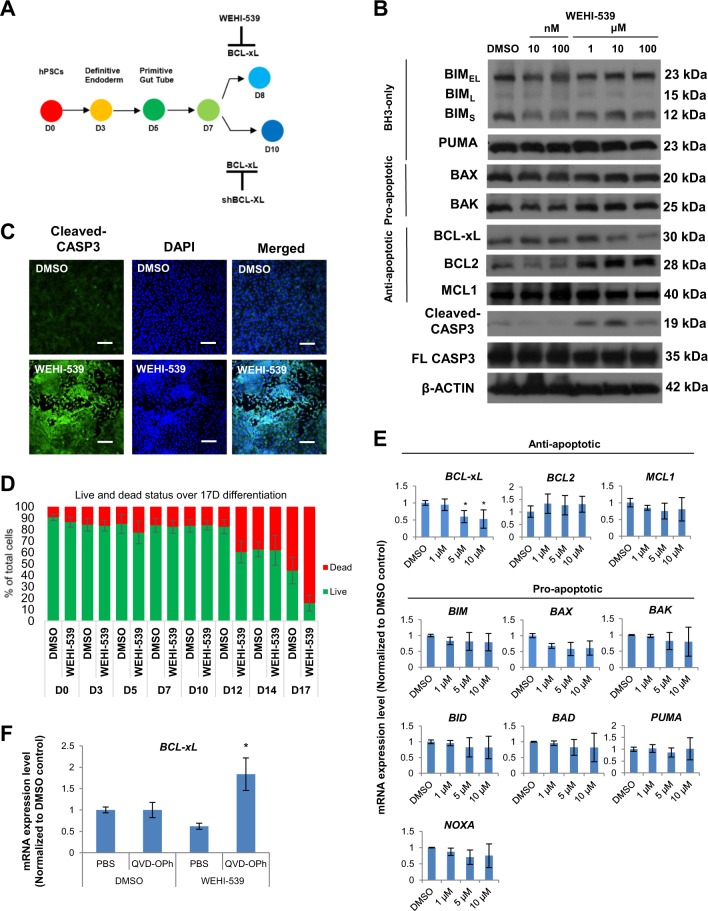
Inhibition of BCL-xL protein function increases apoptosis from D7 onwards. **a** Schematic showing the usage of WEHI-539 or sh*BCL2L1* to inhibit BCL-xL at D7 during pancreatic specification. **b** Western blot showing the expression of BCL-2 proteins upon treatment with WEHI-539 on D7 cells. **c** Immunofluorescence staining for cleaved CASP3 protein on cells treated with DMSO or WEHI-539. Scale bar represents 100 μm. **d** LIVE/DEAD Viability/Cytotoxicity to quantify the percentage of live and dead cells after treatment with DMSO or WEHI-539 on all eight timepoints during pancreatic differentiation. **e** Expression of anti-apoptotic and pro-apoptotic gene transcripts upon treatment with WEHI-539 on D7 cells. **f** Expression of *BCL-xL* gene transcripts upon treatment with WEHI-539 and QVD-OPh on D7 cells. Error bars indicate standard deviation of three biological replicates undergoing independent differentiations. Asterisk (*) indicates *P* < 0.05 compared to DMSO control by one-way ANOVA. A representative of at least two independent experiments is shown. “See also Fig. S2”.

Since BCL-xL expression started to increase on D7 of our differentiation, we decided to assess the importance of BCL-xL protein during early pancreatic specification at this timepoint (Fig. 2a). We used a specific and high affinity BCL-xL inhibitor, WEHI-539, that interacts with the binding groove of BCL-xL (but does not act on BCL2) to inhibit its anti-apoptotic activity^13^. An increasing dose of WEHI-539 from 10 nM to 100 μM on D7 cells resulted in a decrease in BCL-xL protein and, a reciprocal increase in BCL2 and cleaved CASP3 protein levels (Fig. 2b), suggesting that BCL-xL is indeed involved in suppressing cleaved CASP3 in the pancreatic progenitors to prevent cell death. Importantly, pro-apoptotic BAK protein levels remained high despite an increase in BCL2 protein expression, indicating that BCL-xL (but not BCL2) is the main anti-apoptotic protein that promotes cell survival during pancreatic specification. The increase in cleaved CASP3 expression upon inhibition of BCL-xL with WEHI-539 was confirmed via immunostaining analyses (Fig. 2c). We also assessed the percentage of live versus dead adherent cells across eight timepoints and our data showed that increased cell death was observed on D12 and D17 upon WEHI-539 treatment (Fig. 2d), suggesting that the later stage of differentiating pancreatic progenitors could be more reliant on BCL-xL for survival. QPCR analyses then demonstrated that 5–10 μM WEHI-539 treatment resulted in decreased *BCL2L1* transcript levels but not any of the other BCL-2 family members that we evaluated (Fig. 2e). We then co-treated D7 cells with WEHI-539 and QVD-OPh, an irreversible pan-caspase inhibitor, to block WEHI-539-induced cell death. Upon addition of QVD-OPh, we observed a rescue of *BCL2L1* transcript expression in the WEHI-539-treated samples (Fig. 2f), suggesting that cells with high *BCL2L1* expression were indeed selectively killed when BCL-xL was inhibited by WEHI-539 treatment.

### RNA-Seq analyses reveal that the inhibition of BCL-xL function kills the more differentiated pancreatic progenitors that are dependent on BCL-xL for survival, leaving the less differentiated cells expressing lower level of pancreatic genes

**Fig. 3:**
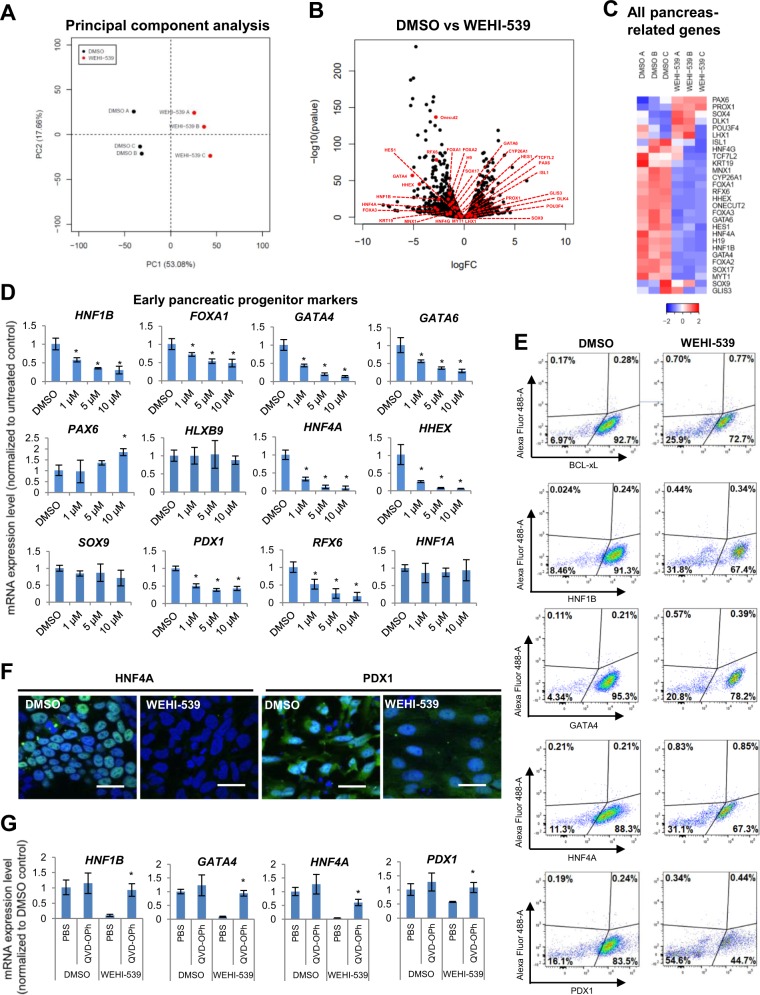
RNA-Seq analyses reveal that the inhibition of BCL-xL function decreases the expression of pancreatic genes. **a** PCA or **b** gene-expression volcano plot of cells treated with DMSO or WEHI-539. **c** Hierarchical clustering heatmap analysis of pancreatic genes (red dots) in D7 cells treated with DMSO or WEHI-539. Colors in the heat map depict gene expression in units of SD from the mean across all samples (upregulation in red, downregulation in blue). **d** Expression of pancreatic gene transcripts upon treatment with WEHI-539 on D7 cells. **e** FACS analysis for BCL-xL, HNF1B, GATA4, HNF4A, and PDX1 proteins in cells treated with DMSO or WEHI-539. **f** Immunofluorescence staining for HNF4A and PDX1 proteins in cells treated with DMSO or WEHI-539. Scale bar represents 50 μm. **g** Expression of *HNF1B*, *GATA4*, *HNF4A*, and *PDX1* gene transcripts upon treatment with WEHI-539 and QVD-OPh on D7 cells. Error bars indicate standard deviation of three biological replicates undergoing independent differentiations. Asterisk (*) indicates *P* < 0.05 compared to DMSO control by one-way ANOVA. A representative of at least two independent experiments is shown. “See also Figs. S2 and S3”.

To evaluate the global impact of BCL-xL inhibition on D7 cells, we performed genome-wide RNA-Seq analyses (Table S1). Principal component analyses indicated that triplicates of DMSO and WEHI-539-treated cells (dead cells had been washed away) clustered distinctly into two groups, suggesting that the inhibition of BCL-xL had an indirect impact on the overall transcriptome profile of these D7 cells (Fig. 3a). We then scrutinized many of the well-established early pancreatic markers and surprisingly found that they were mostly downregulated in the WEHI-539-treated cells as visualized from the volcano plot (Fig. 3b) and heatmap (Fig. 3c; FC > 1.5; *P* < 0.05). To confirm these observations, we performed QPCR analyses and validated a distinct dose-dependent downregulation of early pancreatic markers including that of *HNF1B*, *FOXA1*, *GATA4*, *GATA6, HNF4A, HHEX, PDX1*, and *RFX6* when WEHI-539 was increased from 1 to 10 μM (D7–D8 treatment; dead cells had been washed away) (Fig. 3d). However, the inhibition of BCL-xL function did not have a global impact on pancreatic gene expression as evidenced from the increase in *PAX6* gene expression and a lack of change in *HLXB9*, *SOX9* or *HNF1A* gene expression (Fig. 3d). We then confirmed the downregulation of several pancreatic genes at the protein level via FACS quantification (Fig. 3e; isotype control not shown) and immunostaining analyses (Fig. 3f). Next, we demonstrated that upon blocking apoptosis with a pan-caspase inhibitor, QVD-OPh, there was increased survival of cells as compared to those treated with WEHI-539 only (Fig. S2a). We also observed a rescue of *BCL-xL/BCL2L1* (Fig. 2f)*, HNF1B, GATA4, HNF4A*, and *PDX1* transcript levels as compared to WEHI-539-treated cells (Fig. 3g). The results suggest that when apoptosis is blocked, the more differentiated pancreatic progenitors may have survived, leading to rescued levels of *BCL2L1, HNF1B, GATA4, HNF4A*, and *PDX1* transcript expression.

To be certain of the importance of BCL-xL during the differentiation of pancreatic progenitors from hPSCs, we then cloned two independent shRNA constructs targeting *BCL2L1* transcript, generated lentiviruses, and transduced D7 cells to knockdown *BCL2L1* before analyzing them on D10 (Fig. 2a). Upon successful knock down of *BCL2L1* transcripts (Fig. S2b), we observed that the sh*BCL2L1* pancreatic progenitors experienced higher cell death and appeared morphologically different (Fig. S2c). QPCR analyses further confirmed that many pancreatic genes such as *HNF1B*, *FOXA1*, *GATA4*, *GATA6*, *HNF4A, HHEX, PDX1*, and *RFX6* were similarly downregulated in sh*BCL2L1* pancreatic progenitors as compared to control sh*SCR* (scrambled; non-targeting) cells (Fig. S2d). We noted slight discrepancies in the gene expression of *PAX6*, *HLXB9*, and *HNF1A* between one day of WEHI-539 treatment and 3 days of shRNA-mediated knockdown (Fig. S2d). We postulate that these differences could be attributed to differences between active inhibition of BCL-xL protein function and an actual decrease in total *BCL2L1* transcript or BCL-xL protein availability. Nonetheless, pancreatic progenitor gene *SOX9* remained unchanged in both treatment conditions. Together, our results highlight an important role for BCL-xL in establishing the survival and identity of well-differentiated pancreatic progenitors. Upon the loss of BCL-xL expression and function, the less differentiated pancreatic progenitors which are not so dependent on BCL-xL for survival, remain and express lower level of pancreatic genes.

### Wnt signaling that may play a role in pancreatic specification is perturbed in less differentiated pancreatic progenitors that are not reliant on BCL-xL for survival

**Fig. 4:**
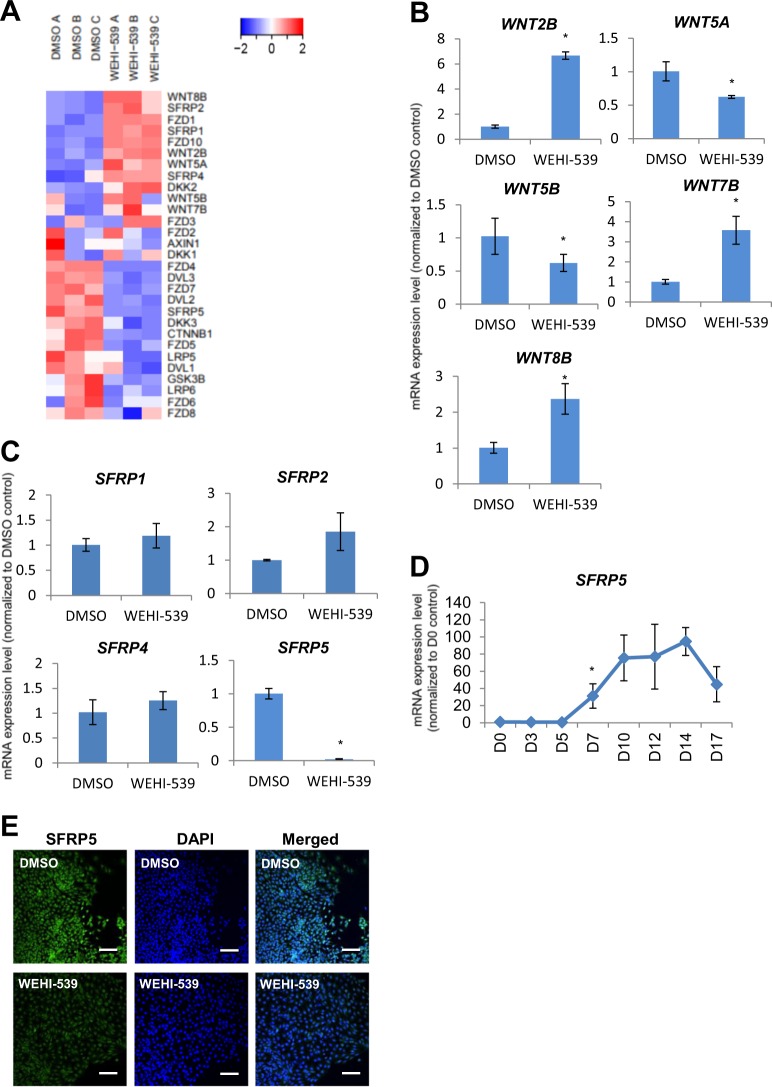
Inhibition of BCL-xL function perturbs Wnt signaling that may play a role in pancreatic specification. **a** Hierarchical clustering heatmap analysis of Wnt signaling-associated genes in D7 cells treated with DMSO or WEHI-539. Colors in the heat map depict gene expression in units of SD from the mean across all samples (upregulation in red, downregulation in blue). Expression of **b** *WNT* or **c** *SFRP* family of transcripts upon treatment with WEHI-539 on D7 cells. **d** Expression of *SFRP5* transcripts over the course of 17D differentiation in H9 hESCs. Error bars indicate standard deviation of three biological replicates undergoing independent differentiations. Asterisk (*) indicates *P* < 0.05 compared to DMSO control or D0 by one-way ANOVA. A representative of at least two independent experiments is shown. **e** Immunofluorescence staining for SFRP5 protein on cells treated with DMSO or WEHI-539. Scale bar represents 100 μm. “See also Fig. S3”.

Upon scrutinization of the RNA-Seq datasets, we also observed numerous genes in the Wnt signaling pathway that were differentially expressed when BCL-xL function was inhibited with WEHI-539 (Fig. 4a). Wnt signaling has been known to be involved in pancreas formation^14^ and is important for pancreatic growth^15^. Amongst the Wnt ligands, *WNT2B*, *WNT5A*, *WNT5B*, *WNT7B*, and *WNT8B* gene expression was perturbed (Fig. 4b). Among the secreted Frizzled-related proteins (SFRPs), which are Wnt antagonists, *SFRP5* was found to be downregulated in the less differentiated pancreatic progenitors (Fig. 4c). Interestingly, *SFRP5* transcripts were found to be increasingly expressed during pancreatic differentiation from hPSCs (Fig. 4d), suggesting its relevance in pancreatic progenitor formation. In addition, SFRP5 protein was found to be expressed in D7 pancreatic progenitors but its expression was diminished upon the inhibition of BCL-xL protein function (Fig. 4e). In view that Sfrp5 has been suggested to be important for pancreatic bud formation in the *Xenopus*^16^ and also for gastrointestinal organogenesis in the zebrafish^17^, this decrease in SFRP5 expression could partly contribute to the broad downregulation of pancreatic marker gene expression upon loss of BCL-xL protein function.

### Less differentiated pancreatic progenitors have decreased level of metabolic activity during pancreatic differentiation from hPSCs

Besides downregulation of pancreatic gene expression, our RNA-Seq and gene ontology analyses also revealed a downregulation of metabolic processes in the less differentiated pancreatic progenitors (Fig. S3a). This was further evidenced via heatmap analyses, demonstrating a distinct cluster of metabolic-related genes being broadly downregulated in less differentiated pancreatic progenitors (Fig. S3b, c). In view that metabolic processes broadly involve oxygen consumption and carbon dioxide production, we decided to evaluate glycolysis and oxidative phosphorylation processes in D7 pancreatic progenitors.

**Fig. 5:**
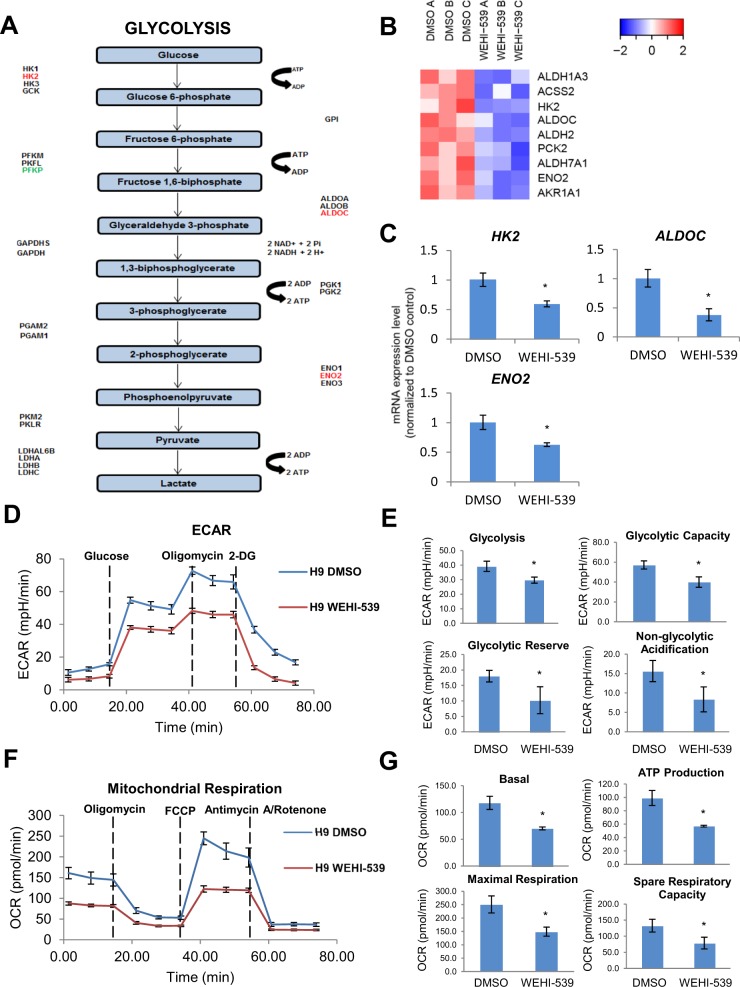
BCL-xL function contributes to metabolic processes that occur during pancreatic specification. **a** Graphical representation of genes perturbed in the glycolysis pathway (upregulation in green and downregulation in red). **b** Hierarchical clustering heatmap analysis of metabolic genes in D7 cells treated with DMSO or WEHI-539. Colors in the heat map depict gene expression in units of SD from the mean across all samples (upregulation in red and downregulation in blue). **c** Expression of metabolic gene transcripts upon treatment with WEHI-539 on D7 cells. Error bars indicate standard deviation of three biological replicates undergoing independent differentiations. Asterisk (*) indicates *P* < 0.05 compared to DMSO control. A representative of at least two independent experiments is shown. **d** Glycolysis stress test and **e** individual component graphs of glycolysis, glycolytic capacity, glycolytic reserve, and non-glycolytic acidification in D7 cells treated with DMSO or WEHI-539. **f** Mitochondrial respiration and **g** individual component graphs of basal mitochondrial respiration, ATP production, maximal respiration, and spare respiratory capacity in D7 cells treated with DMSO or WEHI-539. Error bars indicate standard deviation of eight replicates. Asterisk (*) indicates *P* < 0.05 compared to DMSO control. A representative of at least two independent experiments is shown. “See also Figs. S3 and S4”.

KEGG pathway mapping and heatmap analyses on multiple genes involved in glycolysis first reflected that most of them were downregulated in the less differentiated pancreatic progenitors (Fig. 5a, b). QPCR analyses further confirmed that glycolytic genes such as *HK2*, *ALDOC* and *ENO2* were downregulated in the less differentiated pancreatic progenitors (Fig. 5c). Pan-caspase inhibitor QVD-OPh was then added to block WEHI-539-induced apoptosis and we observed a rescue of *HK2, ALDOC* and *ENO2* transcript levels (Fig. S3d). When glycolysis stress test was performed on D7 pancreatic progenitors using the Seahorse bioenergetics assays, we detected lower extracellular acidification rate (from glycolysis-derived lactate) in both the less differentiated pancreatic progenitors from H9 hESCs (Fig. 5d) and iAGb hiPSCs (Fig. S4a). The rate of glycolysis (basal condition) and maximum glycolytic capacity (upon the injection of mitochondrial ATP synthase inhibitor, oligomycin) were consistently decreased in the less differentiated pancreatic progenitors (Figs. 5d, e, S4a, b). Interestingly, non-glycolytic acidification was also lower in the less differentiated pancreatic progenitors (Figs. 5e and S4b).

Next, we performed mito stress test to determine the mitochondrial function via oxygen consumption rate given that BCL-xL is localized on the outer mitochondrial membrane. We detected lower basal respiration, ATP production, maximal respiration (facilitated by FCCP that disrupts mitochondrial membrane potential), and spare capacity (difference between maximal and basal respiration) in the less differentiated pancreatic progenitors after the more differentiated D7 pancreatic progenitors that were dependent on BCL-xL for survival were killed by WEHI-539 treatment (Figs. 5f, g and S4c, d). Together, these data indicated that the anti-apoptotic role of BCL-xL in differentiating pancreatic progenitors contributes to their overall metabolic function and profile.

### Perturbation of BCL-xL early on during pancreas specification has detrimental long-term impact on pancreatic beta cell formation

**Fig. 6:**
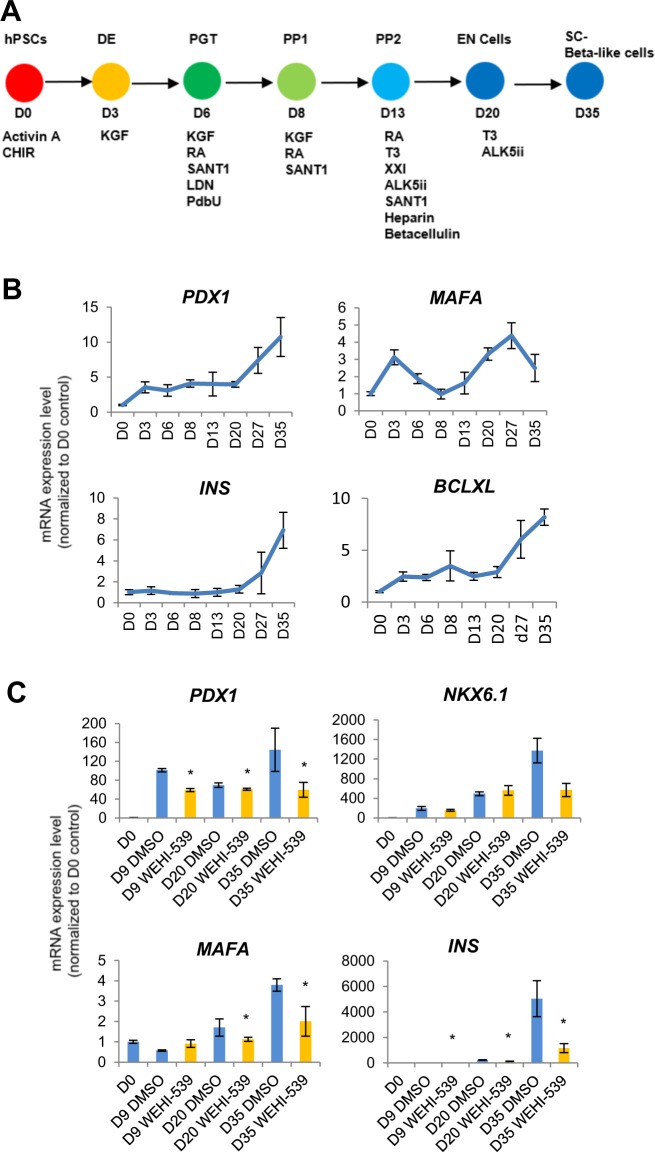
Perturbation of BCL-xL early on during pancreas specification has detrimental long-term impact on pancreatic beta cell formation. **a** Schematic showing 35D differentiation protocol used to differentiate hPSCs into pancreatic beta-like cells. Respective growth factors used are depicted at each time point. **b** Expression of *PDX1*, *MAFA*, *INS*, and *BCL-xL* transcripts over the course of 35D differentiation in H9 hESCs. **c** Expression of *PDX1*, *NKX6.1*, *MAFA*, and *INS* transcripts upon treatment with WEHI-539 on D8 cells followed by the completion of 35D differentiation in H9 hESCs. Error bars indicate standard deviation of three biological replicates undergoing independent differentiations. Asterisk (*) indicates *P* < 0.05 compared to DMSO control by one-way ANOVA. A representative of at least two independent experiments is shown.

Last but not least, to determine the relevance of BCL-xL during early pancreatic progenitor formation on pancreatic beta-like cells, we further differentiated hPSCs into INS^+^ pancreatic beta-like cells adapted from an established protocol (Fig. 6a)^18^. QPCR analyses confirmed that key pancreatic beta cell genes *PDX1*, *MAFA*, and *INS* increased in gene expression over the course of 35 days (Fig. 6b). While *BCL2L1* gene expression was higher on D8 as compared to D0, interestingly, its expression continued to increase as *INS* transcripts were being expressed (Fig. 6b), suggesting its involvement throughout the course of beta cell differentiation.

**Fig. 7:**
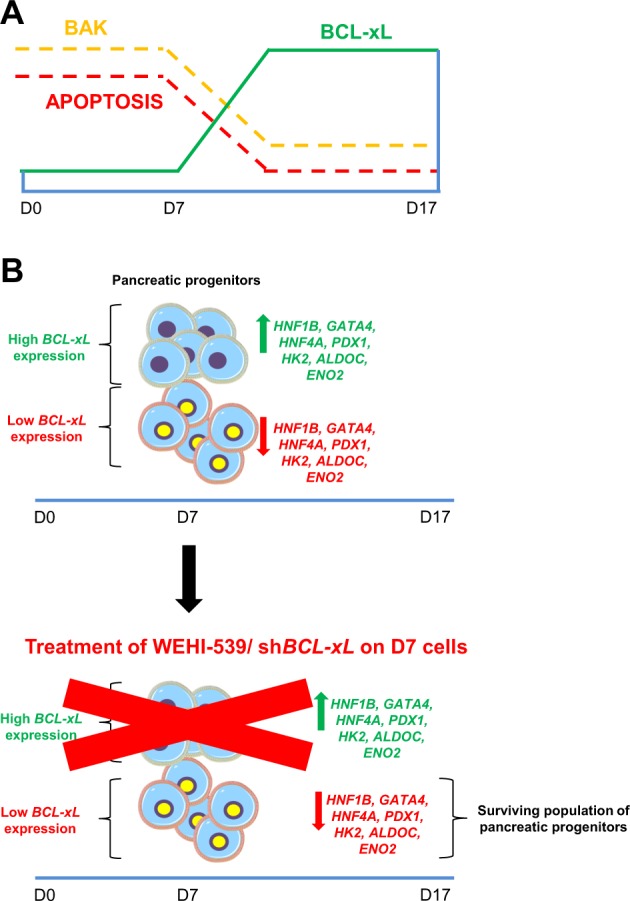
Schematic of BCL-xL and BAK playing reciprocal roles during pancreatic specification. **a** Inverse relationship between BCL-xL and BAK proteins contributing to pancreatic specification. **b** Inhibition of BCL-xL leads to an indirect decrease in pancreatic and glycolytic gene expression due to the loss of more differentiated pancreatic progenitors that are more dependent on BCL-xL for survival.

Therefore, we treated D8 pancreatic progenitors with BCL-xL inhibitor WEHI-539 and followed the cells as differentiation progressed to D35 pancreatic beta-like cells (Fig. S5). QPCR analyses revealed that the inhibition of BCL-xL function on D8 killed the BCL-xL-dependent pancreatic progenitors and led to a population of less differentiated pancreatic progenitors, ultimately leading to a decrease in key pancreatic beta cell gene expression including that of *PDX1*, *MAFA*, and *INS* (Fig. 6c). Together, these data indicated that BCL-xL played an important anti-apoptotic role during early human pancreatic differentiation which had a long-term impact on the eventual pancreatic beta cell formation (Fig. 7a, b).

## Discussion

The cell-type and developmental stage-specific effects of BCL2^19–21^, BCL-xL^22–24^, and other BCL-2 proteins have often been shown, both in genetic mouse models and human cells. Although both BCL-xL and BCL2 have been reported to be important in the pancreas, during the state of pancreatitis^25^, here, we report a unique reciprocal relationship between BCL-xL (but not BCL2) and BAK during early human pancreatic differentiation.

In undifferentiated hPSCs, BAK, which has been shown to be essential for apoptosis^26,27^, is initially expressed at high levels. However, as pancreatic specification occurs from D7 onwards, BAK protein expression decreases, in concert with an increase in the protein expression of BCL-xL and a decrease in downstream apoptotic cleaved caspase 3. The association of BCL-xL but not BCL2 with BAK has also been reported to keep apoptosis in check^28,29^, albeit not specifically during pancreatic development. This is consistent with our findings that the compensatory increase in BCL2 protein expression upon BCL-xL inhibition with the highly specific WEHI-539 is insufficient to curb the BAK-mediated increase in cleaved caspase 3^13^ and the triggering of the caspase cascade. *BAK* transcript levels did not change upon BCL-xL inhibition possibly because changes are only evident at the protein level. Additional experiments performed in undifferentiated hPSCs have confirmed that there is no direct inhibitory relationship between BCL-xL and BAK expression levels as BAK protein level remained constant when BCL-xL was overexpressed in hPSCs. Apart from contributing to the survival of pancreatic progenitors, BCL-xL has also been reported to be vital in ensuring the survival of neurons in the developing brain and spinal cord^22^.

Intriguingly, we found that the downregulation of BCL-xL expression and function resulted in an indirect overall decrease in early pancreatic gene and protein expression due to survival of only the less differentiated pancreatic progenitors. While BCL-xL is known to be dispensable during rodent beta cell development, it is important for protection against apoptotic stimuli in mature beta cells^23^. The inhibition of BCL-xL with WEHI-539 could have resulted in higher death in cells that expressed high level of pancreatic markers. These more differentiated cells could be more sensitive to apoptosis and are dependent on anti-apoptotic BCL-xL for survival. This leaves behind less differentiated pancreatic progenitors that expressed lower levels of pancreatic genes. Therefore, we propose that BCL-xL plays an important permissive role in human pancreatic specification that is ultimately crucial for proper beta cell differentiation from hPSCs. While we found BCL-xL to play this role during human pancreatic specification possibly by supporting the survival of differentiating pancreatic progenitors, we do not rule out the importance of BCL2 or other anti-apoptotic proteins in this process. When WEHI-539-induced apoptosis was blocked by the addition of pan-caspase inhibitor QVD-OPh, we observed a rescue in the transcript levels of *BCL2L1*, *HNF1B*, *GATA4*, *HNF4A*, and *PDX1*, indicating that the more differentiated pancreatic progenitors may have survived, leading to the observed rescue phenotype.

Upon inhibition of BCL-xL function in D7 pancreatic progenitors, we found perturbations in Wnt signaling molecules and a distinct downregulation of SFRP5 protein. Given the known role of Wnt signaling in pancreas development^14,15^ and the importance of Sfrp5 in pancreatic organogenesis in both the *Xenopus*^16^ and zebrafish^17^, we postulate that this could partly contribute to the indirect effects of loss of *BCL2L1*/BCL-xL gene and protein function on the overall downregulation of pancreatic marker gene expression in the remaining pancreatic progenitors. However, we do not rule out other possible mechanisms as well.

Interestingly, the inhibition of BCL-xL function also indirectly affected metabolic processes in the residual pancreatic progenitors possibly due to the loss of cells that were more dependent on BCL-xL for survival. Since cellular metabolism can affect differentiation and development^30^, we posit that this perturbation at the mitochondrial level also partly contributes to the mechanistic link between BCL-xL function and pancreatic development. BCL-xL has been reported to be directly involved in mitochondrial energetic capacity that is necessary for cell survival^31^. Inhibition of WEHI-539-induced apoptosis demonstrated enhanced viability of pancreatic progenitors as evident by both the rescue of pancreatic and metabolic gene expression. Therefore, the loss of BCL-xL function could be more detrimental to pancreatic progenitors that are more differentiated and more dependent on BCL-xL for survival. In neurons, BCL-xL deficiency results in a defect in the control of mitochondrial membrane potential, giving rise to a leaky inner mitochondrial membrane^31^. These BCL-xL-deficient cells eventually depolarize and die off as they are unable to maintain a normal membrane potential. Through interacting with the mitochondrial F_1_F_0_ ATP synthase, BCL-xL was also shown to regulate the metabolic efficiency of neurons^24^. These studies highlight the non-canonical role of BCL-xL in the regulation of metabolism in different cell types.

Together, we report a previously unappreciated role for BCL-xL in promoting the survival of differentiating pancreatic progenitors from hPSCs. Modulation of the expression level of BCL-xL during human pancreatic specification from hPSCs could possibly be a means to improve the survival and robustness of pancreatic progenitors that ultimately determine pancreatic beta cell mass and function.

## Method

### hPSC culture, 17D differentiation, and 35D differentiation

Undifferentiated hPSCs were maintained in DMEM/F-12 with 15 mM HEPES (STEMCELL Technologies), 20% KnockOut^™^ serum replacement (KOSR) (Gibco), l-Glutamine (Sigma), NEAA (Life Technologies) and supplemented with 10 ng/ml FGF2 (Miltenyi Biotec) in 5% CO_2_ and 100% humidity. hPSCs media were replaced every 24 h. hPSCs were manually split and seeded on irradiated CF-1 mouse embryonic fibroblasts (MTI-GlobalStem) once a week.

iAGb was cultured in feeder-free condition using TeSR™-E8™ basal medium and TeSR^™^-E8^™^ 25× Supplement (STEMCELL Technologies). For 17D differentiation setup, the cells were processed as according to the 17D differentiation protocol described below.

293FT cells were cultured in DMEM High glucose (Life Technologies) supplemented with 10% Fetal bovine serum (South America Hyclone) and 1% nonessential amino acid NEAA (Invitrogen).

All cell lines are screened routinely for mycoplasma contamination and are declared to be mycoplasma-free.

### 17D differentiation

At D-2, confluent hPSCs in 10 cm dish were washed with sterile phosphate-buffered saline (PBS) and incubated with Dispase (STEMCELL Technologies) and Collagenase IV (Life Technologies) for 5 min. The cells were then washed with sterile PBS, scored and passed through 70 μM cell strainer. For each 10 cm plate, 6 ml hPSC medium was used to flush the plate and cell suspension collected. The cell suspension was then dispensed into a 6 well plate and left to incubate for 48 h at 5% CO_2_ and 100% humidity.

Cells were differentiated 2 days later in RPMI-1640/2% B-27 (no vitamin A; serum-free chemically defined medium (Gibco) supplemented with 1% GlutaMAX Supplement (Invitrogen), 1% MEM nonessential amino acids NEAA (Invitrogen), 0.1% β-mercaptoethanol (Gibco). The component of each respective media is as follow: D0: 100 ng/ml Activin A (R&D System). 3 μM CHIR9021 (Tocris), 10 μM LY294002 (LC Labs). D3: 50 ng/ml Activin A (R&D Systems). D5: 50 ng/ml FGF2 (Miltenyi Biotec) + 3 μM RA (Wako) + 10 mM Nic (Sigma). D7: 50 ng/ml FGF2 (Miltenyi Biotec) + 3 μM RA (Wako) + 10 mM Nic (Sigma). D10: 50 ng/ml FGF2 (Miltenyi Biotec) + 3 μM RA (WAKO) + 10 mM Nic (Sigma) + 20 μM DAPT (Abcam). D12: 50 ng/ml FGF2 + 3 μM RA (WAKO) + 10 mM Nic (Sigma) + 20 μM DAPT (Abcam). D14: 50 ng/ml FGF2 (Miltenyi Biotec) + 10 mM Nic (Sigma) + 20 μM DAPT (Abcam). The cells were then harvested at each respective time-point for RNA and protein analysis.

### 35D differentiation

At D-2, confluent hPSCs in 10 cm dish were washed with sterile PBS and incubated with 3 ml of TrypLE (Life Technologies) for 3 min. The cells were then washed with sterile PBS. 5 ml of DMEM/F12 media were then dispensed into the dish to flush the cells and the cell solution was filtered through 70 μM filter. The cells were centrifuged at 1200 rpm for 5 min and seeded at 1 million cell/ml in a low attachment plate. The plate was left to incubate for 48 h at 5% CO_2_ and 100% humidity in a shaker at 80 rpm. The cells were differentiated using the growth factors on the respective days. D0: Activin A 100 ng/ml (R&D system) + CHIR9021 (Tocris). D1: Activin A 100 ng/ml (R&D system). D3: FGF7 50 ng/ml (Mitenyi Biotec). D5: FGF7 50 ng/ml (Mitenyi Biotec). D6: FGF7 50 ng/ml (Mitenyi Biotec) + RA 2 μM (WAKO) + Sant1 0.25 μM (Santa Cruz) + PDBu 500 nM + LDN 200 nM. D7: FGF7 50 ng/ml (Mitenyi Biotec) + RA 2 μM (WAKO) + Sant1 0.25 μM (Santa Cruz) + PDBu 500 nM. D8: FGF7 50 ng/ml (Mitenyi Biotec) + RA 100 nM (WAKO) + Sant1 0.25 μM (Santa Cruz). D10: FGF7 50 ng/ml (Mitenyi Biotec) + RA 10 nM (WAKO) + Sant1 0.25 μM (Santa Cruz). D12: FGF7 50 ng/ml (Mitenyi Biotec) + RA 100 nM (WAKO) + Sant1 0.25 μM (Santa Cruz). D13: RA 100 nM (WAKO) + Sant1 0.25 μM (Santa Cruz) + XXI 1 μM (Millipore) + Alk5ill 10 μM (ENZO) + T3 1 μM (Millipore) + Betacellulin 20 ng/ml (Cell Signaling). D15: RA 100 nM (WAKO) + Sant1 0.25 μM (Santa Cruz) + XXI 1 μM (Millipore) + Alk5ill 10 μM (ENZO) + T3 1 μM (Millipore) + Betacellulin 20 ng/ml (Cell Signaling). D17: RA 25 nM (WAKO) + XXI 1 μM (Millipore) + Alk5ill 10 μM + T3 1 μM (Millipore) + Betacellulin 20 ng/ml (Cell Signaling). D19: RA 25 nM (WAKO) + XXI 1 μM (Millipore) + Alk5ill 10 μM (ENZO) + T3 1 μM (Millipore) + Betacellulin 20 ng/ml (Cell Signaling). D20: Alk5ill 10 μM (ENZO) + T3 1 μM (Millipore). D22: Alk5ill 10 μM + T3 1 μM (Millipore). D24: Alk5ill 10 μM (ENZO) + T3 1 μM (Millipore). D26: Alk5ill 10 μM + T3 1 μM (Millipore). D28: Alk5ill 10 μM (ENZO) + T3 1 μM (Millipore). D30: Alk5ill 10 μM (ENZO) + T3 1 μM (Millipore). D32: Alk5ill 10 μM (ENZO) + T3 1 μM (Millipore). D34: Alk5ill 10 μM (ENZO) + T3 1 μM (Millipore). The cells were then harvested at each respective time-point for RNA analysis.

### Cell culture treatment

H9 cells were differentiated using 17D differentiation protocol to D7 and treated with 10 μM of WEHI-539 (ApexBio), an inhibitor of BCL-xL. On D8, the cells were harvested for QPCR, western blot and immunostaining. 10 µM broad-spectrum caspase inhibitor QVD-OPh (Cayman Chemical) was used to inhibit apoptosis.

### RNA extraction and RT-PCR

RNA was extracted from the cells using RNA isolation Nucleospin^®^ RNA (Macherey-Nagel). 350 μl of RA1 buffer was added to each well of a 12-well plate. Cell homogenates were transferred to spin columns and processed as according to the manufacturer’s instructions. Purified RNA was quantified using the NanoDrop 1000 spectrophotometer (Thermo Fisher Scientific). One microgram of RNA was converted to cDNA using High capacity cDNA Reverse Transcription Kit (Applied Biosystems). For 1 reaction, the following were mixed, 2 μl 10× RT buffer, 0.8 μl 25× dNTP mix (100 mM), 2 μl 10× RT random primers, 1 μl Multiscribe^TM^ reverse transcriptase. The mixture was topped up with respective amount of nuclease-free water to 20 μl. The protocol is as follows: 25 °C for 10 min, 37 °C for 120 min, 85 °C for 5 min, and 4 °C forever.

### Quantitative real-time PCR (QPCR)

QPCR was performed on the CFX384 Touch^™^ Real-Time PCR Detection System with iTaq^™^ *Universal SYBR*^*®*^ *Green Supermix* (Bio-Rad). For 1 QPCR reaction, the following were mixed. Five microilitre SYBR Green Supermix, 300 nM Forward primer, 300 nM Reverse primer, 1.9 μl nuclease-free water, 2.5 μl cDNA (2.5 ng/μl) to a final volume of 10 μl. The samples were loaded in duplicate on a 384-well plate (Applied Biosystems). The thermal cycling condition was as follow: 95°C for 3 min, 9 °C for 5 s, 60 °C for 30 s, repeated for 39 cycles and then 65 °C for 30 s, 65 °C for 5 s, and 4 °C forever. Fold changes are normalized to β-actin gene expression and are based on relative expression values calculated using the 2^−ΔΔC(T)^ method. QPCR primers were designed to span exon–exon junction, where possible, using Primer-BLAST (NCBI). Sequences of primers are listed in Table S2.

### SDS-PAGE/Western blot

Cells were washed with PBS and treated with Trypsin for 5 min at 37 **°**C. Dislodged cells were neutralized with MEF media and centrifuged at 1500 rpm for 5 min to obtain the cell pellet. Cell pellet were lysed in M-PER mammalian protein extraction reagent (Thermo Fisher Scientific) in the presence of protease and phosphatase inhibitors (Sigma). Protein lysates were then quantified using BCA assay kit (Thermo Fisher Scientific). Sodium dodecyl sulfate polyacrylamide gel electrophoresis (SDS-PAGE) was performed using the Mini-PROTEAN Tetra Cell system (Bio-Rad) at 150 V for 1 h. Forty microgram of proteins were then transferred to PVDF membranes (Bio-Rad) at 100 V for 1 h. The blots were then blocked for 1 h in 5% milk (Anlene nonfat milk) and probed with the respective primary antibodies and secondary antibodies, Primary antibodies used in this study: Mouse monoclonal anti-β-actin (1:10,000 Sigma, A5541), Rabbit polyclonal anti-BAX (1:1000, Cell Signaling Technologies, #2772 S), Rabbit polyclonal anti-BAK (1:1000, Cell Signaling Technology, #3814S), Rabbit polyclonal anti-BCL-xL (1:1000, Cell Signaling Technology #2762S), Rabbit polyclonal anti-BCL2 (1:1000, Cell Signaling Technology #2876), Rabbit polyclonal anti-BIM (1:1000, Cell Signaling Technology #2819S), Rabbit polyclonal anti-Caspase 3 (1:1000, Cell Signaling Technology, #9662S), Rabbit polyclonal anti-MCL1 (1:1000, Cell Signaling Technology, #4572), Rabbit polyclonal anti-PUMA (1:1000, Cell Signaling Technology #4976). Secondary used in this study: Goat anti-rabbit IgG HRP (1:10,000, Santa Cruz, sc-2004), Goat anti-mouse IgG HRP (1:10,000, Santa Cruz, sc-2005). Chemiluminescent signals were detected using Super Signal^TM^ West Dura Extended Duration substrate (Thermo Fisher Scientific).

### Immunostaining

At the end of each differentiation time-point, cells on coverslips (Marienfeld) were washed with DPBS and fixed with 4% paraformaldehyde for 20 min at room temperature. Cells were blocked with DPBS containing 5% donkey serum and 0.1% Triton X-100 at 4 °C for 1 h before overnight incubation with primary antibodies at 4 °C. Primary antibodies used in this study: Rabbit monoclonal anti-BCL-xL (1:1000, Abcam, Ab178844), Rabbit monoclonal anti-BCL2 (1:150, Abcam, Ab182858), Mouse monoclonal anti-BAK (1:1000, Abcam, Ab104124), Mouse monoclonal anti-GATA4 (1:1000, Thermo Fisher Scientific, MA5-15532), Goat polyclonal anti-HNF1β (1:100, Abcam, Ab59118), Rabbit monoclonal anti-HNF4α (1:1000, Cell Signaling Technology #3113), Goat polyclonal anti-PDX1 (1:20, R&D Systems, Af2419), Rabbit polyclonal anti-Caspase 3 (1:200, Abcam, Ab13847), cells were incubated in the dark with secondary antibodies diluted in DPBS containing 0.1% Triton X-100 for 1 h at 4 °C before staining with DAPI (Sigma) for 20 min at 4 °C. Secondary antibodies used in this study: Alex Fluor Plus 647 Donkey Anti-Mouse IgG (H + L) (1:500, Invitrogen, Ab32787), Alexa Fluor 488 Donkey Anti-Rabbit IgG (H + L) (1:500, Invitrogen, A21206), Alex Fluor 488Donkey Anti-Goat IgG (H + L) (1:500, Thermo Fisher Scientific, A11055), Alex Fluor 488 Donkey Anti-Mouse (H + L) (1:500, Thermo Fisher Scientific, A21202). The coverslips were mounted onto SuperFrost Plus™ Adhesion slides (Thermo Fisher Scientific) using DAKO mounting medium (DAKO). Confocal images were acquired with the Olympus FV1000 inverted confocal microscope using the Olympus Fluoview v3.1 software. Brightfield images were acquired with the Axiovert 200 M inverted microscope using the Axiovision LE software version 4.8.2.

### Live and dead cell assay

hPSCs were differentiated to D17 and for each of the eight timepoints during pancreatic differentiation, the differentiating cells were treated with DMSO or WEHI-539. Prior to staining, the cells were washed twice with DPBS. The LIVE/DEAD^™^ Viability/Cytotoxicity Kit (Thermo Fisher Scientific) was allowed to thaw to room temperature. 2 µl EthD-1: 0.5 µl CalceinAM were added to 1 ml of DPBS. For each well of 12-well plate, 90 µl of the staining solution was used and left to incubate for 10 min at room temperature. The staining solution was removed and washed with DPBS. Confocal images were acquired with the Olympus FV1000 inverted confocal microscope using the Olympus Fluoview v3.1 software. Brightfield images were acquired with the Axiovert 200 M inverted microscope using the Axiovision LE software version 4.8.2. Stained cells were then counted from an average of at least ten images using ImageJ software.

### Fluorescence-activated cell sorting (FACS)

Differentiated cells were harvested by mechanical scraping using a cell scraper and dissociated into single cells following incubation with 0.25% Trypsin/EDTA at 37 °C. Single cells were collected by passing the suspension through a 40 µm cell strainer. Cells were pelleted by centrifugation at 1200 rpm for 5 minutes. Supernatant was aspirated and cells were washed with DPBS, followed by fixation with 4% paraformaldehyde on ice for 1 h. Fixed cells were washed with DPBS before blocking in FACS buffer (5% FBS in DPBS) containing 0.1% Triton X-100 on ice for 1 h. Cells were incubated with primary antibodies for 1 h at 4°C and washed with FACS buffer containing 0.1% Triton X-100 prior to addition of secondary antibodies. Cells were incubated with secondary antibodies in the dark for 1 h at 4 °C. After washing, cells were resuspended in FACS buffer before analysis using the BD^TM^ LSR II Flow Cytometer. Data analysis were performed using the FlowJo 7.0 software.

### Lentiviral-mediated knockdown

shRNAs targeting *BCL2L1* (Sigma Construct: shRNA TRCN0000033500 and TRCN0000033501) were cloned into pLKO.1 vector. Plasmids were extracted using NucleoBond^®^ Xtra Midi (Macherey-Nagel). The plasmids were packaged into virus using HEK293FT cells. Media was changed 24 h after transfection and left to incubate. Media was collected at 48 and 72 h and pooled. The lentivirus in the collected media was concentrated using ultra-clear tubes (Beckman Coulter) and placed in SW28 Ti swinging-bucket aluminum rotor (Beckman Coulter). The SW28 swinging-bucket rotor was loaded in Optima L-100 XP ultracentrifuge (Beckman Coulter) at centrifuged at 23,000 rpm for 1.5 h at 4 °C. 500 µl of DMEM media was used to resuspend the pellet and the suspension was frozen at −80 °C. hPSCs were differentiated to D6, trypsinized into single cells and replated at 400,000 cells per well in a 12-well plate. On D7, the single cells were transduced with the lentivirus with an MOI of 200 in 5 μg/ml polybrene (Millipore) for 24 h. The medium was then changed to normal differentiation media and left to grow for an additional 48 h.

### RNA sequencing and differential expression analysis

Poly-A mRNA was enriched from 1 µg of total RNA with oligo-dT beads (Invitrogen). Up to 100 ng of poly-A mRNA recovered was used to construct multiplexed strand-specific RNA-seq libraries as per manufacturer’s instruction (NEXTflex^TM^ Rapid Directional RNA-SEQ Kit, dUTP-Based, v2). Individual library quality was assessed with an Agilent 2100 Bioanalyzer and quantified with a QuBit 2.0 fluorometer before pooling for sequencing on a HiSeq 2000 (1 × 101 bp read). The pooled libraries were quantified using the KAPA quantification kit (KAPA Biosystems) prior to cluster formation. Adapter sequences and low quality bases in Fastq read sequences were trimmed using Trimmomatic (v.0.33) (parameters: LEADING:3 TRAILING:3 SLIDINGWINDOW:4:15 MINLEN:36). The quality filtered Fastq sequence reads were then aligned to the human genome (hg19) using Tophat (v.2.0.14) (parameters: --no-coverage-search --library-type=fr-firststrand) and annotated with Ensembl gene IDs. The resulting bam files were used to generate feature read counts using the Python package-based htseq-count of HTSeq (v.0.6.1p1) (parameters: default union-counting mode, --stranded=reverse). The read count matrix output from HTSeq was used to perform differential expression analysis using the edgeR package (available in R (v.3.1.3)) in both ‘classic’ and generalized linear model (glm) modes to contrast patient versus control. Procedures described in edgeR documentation were followed to calculate *P* values, false-discovery rate (FDR) adjusted *P* values (*q*-values) and fold-changes. A FDR cutoff of 0.05 was used to filter significantly differentially expressed genes. These genes with Ensembl IDs were mapped to gene symbols.

### Seahorse metabolic flux assay

hESCs were differentiated to D7 using the 17D differentiation protocol and treated with DMSO or WEHI-539 (Apexbio) for 24 h. D8 cells were then trypsinized and 150,000 cells plated per well on Seahorse XF96 cell culture microplates (Agilent). Growth media was changed to Seahorse XF Base Medium (Agilent) supplemented with l-Glutamine (Sigma) and placed in a non-CO_2_ incubator 1 h prior to assay. Glycolysis was measured via the Seahorse XF Glycolysis Stress Test Kit (Agilent) with a Seahorse XF 96 analyzer (Agilent) following the manufacturer’s protocol. Using the same setup, oxidative phosphorylation was measured using Seahorse XF Mito Stress Kit (Agilent).

### Quantification and statistical analysis

Statistical parameters for each experiment, including values of replicates and statistical significance, can be found in the figure legends. For dosage and time-point studies, one-way ANOVA was used to analyze differences in gene expression levels. All other statistical analyses were performed using Student’s *t* test (two-sided; equal variance). *P* values of less than 0.05 were considered significant.

**Key resources table**

**Table:** 

Reagent or resource	Source	Identifier
Antibodies		
Anti-β-actin (Mouse monoclonal)	Sigma	A5441; RRID: AB_476744
Anti-BAX [2D2] Mouse monoclonal	Abcam	Ab77566; RRID: AB_1565901
Anti-BAX (Rabbit polyclonal)	Cell Signaling Technology	#2772S; RRID: AB_10695870
Anti BAK [AT8B4] (Mouse monoclonal)	Abcam	Ab104124; RRID: AB_10712355
Anti-BAK (Rabbit polyclonal)	Cell Signaling Technology	#3814S; RRID: AB_2290287
Anti -BCL-xL [EPR16642] (Rabbit monoclonal)	Abcam	Ab178844; RRID: NA
Anti-BCL-xL (Rabbit polyclonal)	Cell Signaling Technology	#2762S; RRID: AB_10694844
Anti-BCL2 [EPR17509] (Rabbit monoclonal)	Abcam	Ab182858; RRID: AB_2715467
Anti-BCL2 (Rabbit polyclonal)	Cell Signaling Technology	#2876; RRID: AB_2064177
Anti-BIM (Rabbit polyclonal)	Cell Signaling Technology	#2819S; RRID: AB_10692515
Anti-Caspase 3 (Rabbit monoclonal)	Abcam	Ab13847; RRID: AB_443014
Anti-Caspase 3 (Rabbit polyclonal)	Cell Signaling Technology	#9662S; RRID: AB_10694681
Anti-GATA4 [6H10] (Mouse monoclonal)	Thermo Fisher Scientific	MA5-15532; RRID: AB_10989032
Anti-HNF1β (Goat polyclonal)	Abcam	Ab59118; RRID: AB_945772
Anti- HNF4α (Rabbit monoclonal)	Cell Signaling Technology	#3113S; RRID: AB_2295208
Anti-MCL1 (Rabbit polyclonal)	Cell Signaling Technology	#4572; RRID: AB_2281980
Anti-PAX6 [AD1.5] (Mouse monoclonal)	Millipore	Ab570718; RRID: AB_570718
Anti-PDX1 (Goat polyclonal)	R&D Systems	Af2419 RRID: AB_355257
Anti-PUMA (Rabbit polyclonal)	Cell Signaling Technology	#4976; RRID: AB_2064551
Anti-SFRP5 (Rabbit polyclonal)	Abcam	Ab230425; RRID: NA
Donkey Anti-Mouse IgG (H + L) Highly Cross-Adsorbed secondary antibody, Alexa Fluor Plus 647	Invitrogen	Ab32787 RRID: AB_2762830
Goat Anti-Mouse IgG HRP	Santa Cruz	sc-2005; RRID: AB_631736
Donkey Anti-Rabbit IgG (H + L) Alexa Fluor 488	Invitrogen	A21206; RRID: AB_2535792
Goat Anti-Rabbit IgG HRP	Santa Cruz	sc-2004; RRID: AB_631746
Donkey Anti-Goat IgG (H + L) Cross-Adsorbed, Alex Fluor 488	Thermo Fisher Scientific	A11055; RRID: AB_2534102
Donkey Anti-Mouse (H + L) Highly Cross-Adsorbed, Alex Fluor 488	Thermo Fisher Scientific	A21202; RRID: AB_141607
Bacterial and Virus Strains		
N/A	N/A	N/A
Deposited Data		
RNA-Seq	GEO	GSE136064
Experimental Models: Cell Lines		
Mouse: CF-1 mouse embryonic fibroblasts	MTI-GlobalStem	GSC-6001G
HEK293FT	Thermo Fisher Scientific	R70007
Human ESC W09 (Female)	WiCell Research Institute, Inc	15-W0038
Human iPSCs iAGb (Male)	Reprogrammed from fibroblast AG16102, Coriell Institute	N/A
Oligonucleotides		
QPCR primers, shRNA oligos, see Table S2	This paper	N/A
Chemical, peptides and recombinant proteins		
Activin A	R&D system	338-AC-50
ALK5ill	ENZO	ALX-270-445-M001
Ascorbic acid	Sigma	A8960
Betacellulin	Cell Signaling	5235SF
B-27™ Serum Minus Vitamin A	Thermo Fisher Scientific	#12587010
β-mercaptoethanol	Thermo Fisher Scientific	21985-023
Calcium chloride	Sigma	C-5670
CHIR9021	Tocris	4423
CMRL 1066 Supplemented	Mediatech Inc	99-603-CV
CMRL Medium 1066	Life Technologies	11530-037
Collagenase IV	Life Technologies	17104019
DAKO mounting medium	DAKO	S3023
DAPI	Sigma	D9542
DAPT	Abcam	Ab120633
Dispase in DMEM/F12	STEMCELL Technologies	07923
D(+)-Glucose	WAKO	049-31165
Donkey serum	Merck	S-30
DMEM F12 media	Invitrogen	10565042
DMEM/High Glucose Media	Hyclone	SH30243.01
DMSO	Sigma	D2650
FAF-BSA	Proliant	68700
FGF2	Miltenyi Biotec	130-093-838
FGF7	Mitenyi Biotec	130037178
Gelatin (Porcine)	Sigma	G1890
Glutamax^TM^ Supplement	Invitrogen	35050038
HEPES Buffer 1 M	STEMCELL Technologies	07200
HyClone Phosphate Buffered Saline solution	GE Healthcare Life Sciences	SH30256.01
Hyclone^TM^ Fetal bovine serum (South America)	Hyclone	SV30160.03
ITS-X	Life Technologies	51500056
KnockOut™ serum replacement	Gibco	10828028
LDN193189	Sigma	SML0559
L-Glutamine	Sigma	G8540
Lipofectamine^TM^ 2000 Transfection Reagent	Invitrogen	11668027
LY294002	LC labs	L-7962
MCDB131	Life Technologies	10372019
MEM Non-Essential Amino Acids (100×)	Life Technologies	11140-050
M-PER^TM^ Mammalian Protein Extraction Reagent	Thermo Scientific	78501
mTESR^TM^1 Basal Medium	STEMCELL Technologies	85851
NaHCO_3_	Sigma	S5761-500G
QVD-OPh	Cayman Chemical	15260
PDBu	Tocris	4153
Penicillin-Streptomycin	Thermo Fisher Scientific	15140122
Polybrene	Millipore	TR-1003-G
Retinoic Acid	WAKO	186-01114
RPMI-1640	Gibco	11875093
SANT1	Santa Cruz	Sc-203253
TeSR^TM^-E8^TM^ Basal Medium	STEMCELL Technologies	05990
TeSR^TM^-E8^TM^ 25X Supplement	STEMCELL Technologies	05992
TrypLE Express	Life Technologies	12604021
Trypsin-EDTA (0.25%)	Life Technologies	25200056
T3	Millipore	642511
Vitamin B3 (Nicotinamide)	Sigma	N0636-100G
Vitamin C (L-Ascorbic acid)	WAKO	012-04802
WEHI-539	ApexBio	A3935
XXI (Gamma-Secretase Inhibitor)	Millipore	565790
Y-27632	STEMCELL Technologies	72302
Critical commercial assays		
High Capacity cDNA Reverse Transcription Kit	Applied Biosystems	4368813
iTaq™ Universal SYBR^®^ Green Supermix	Bio-Rad	1725124
Lenti-X^TM^ p24 Rapid Titer Kit	Clontech	632200
LIVE/DEAD^®^ Viability/Cytotoxicity Kit	Thermo Fisher Scientific	L-3224
NucleoBond^®^ Xtra Midi	Macherey-Nagel	740410.50
Nucleospin^®^ Plasmid EasyPure	Macherey-Nagel	740727.250
Phusion High-Fidelity DNA Polymerase	Thermo Fisher Scientific	F530S
Pierce^TM^ BCA Protein Assay Kit	Thermo Fisher Scientific	23227
PureLink^TM^ Quick PCR Purification Kit	Invitrogen	K310002
RNA isolation Nucleospin^®^ RNA	Macherey-Nagel	740955.250
Seahorse XF Base Medium	Agilent	102353-100-100
Seahorse XF Glycolysis Stress Test Kit	Agilent	103020-100
Seahorse XF Mito Stress Kit	Agilent	103015-100
SuperSignal^TM^ West Dura Extended Duration Substrate	Thermo Fisher Scientific	34076
Softwares and Algorithms		
AxioVision LE	Zeiss	Version 4.8.2
FlowJo 10	FlowJo	Version 10
Seahorse XFe96 Analyzer	Agilent	S7800B
Seahorse Wave Desktop Software	Agilent	Version 2.6.1
Others		
CFX384^TM^ Real-Time System	Bio-Rad	1855485
Coverslips 18 × 18 mm	Marienfeld	0101030
NanoDrop 1000 spectrophotometer	Thermo Fisher Scientific	V 3.8
Nikon Eclipse Inverted	Nikon	TS-100
Olympus Fluoview 1000 Inverted Confocal	Olympus	FV1000
Optima L-100 XP Ultracentrifuge	Beckman Coulter	L-100 XP
SuperFrost Plus^™^ Adhesion slides	Thermo Fisher Scientific	10149870
SW28 Ti Swinging-Bucket Aluminum Rotor	Beckman Coulter	342207
Ultra-Clear tubes	Beckman Coulter	344058

## Supplementary information

Supplemental Figure 1

Supplemental Figure 2

Supplemental Figure 3

Supplemental Figure 4

Supplemental Figure 5

Table S1

Supplemental Information- clean

## Data Availability

Accession number for RNA-Seq data in GEO is GSE136064.
