# Supplementary figures and images for: BCL-xL/BCL2L1 is a critical anti-apoptotic protein that promotes the survival of differentiating pancreatic cells from human pluripotent stem cells
Source: Cell Death Dis. 2020 May 18;11(5):378. doi: 10.1038/s41419-020-2589-7 (PMC7235254; doi:10.1038/s41419-020-2589-7)

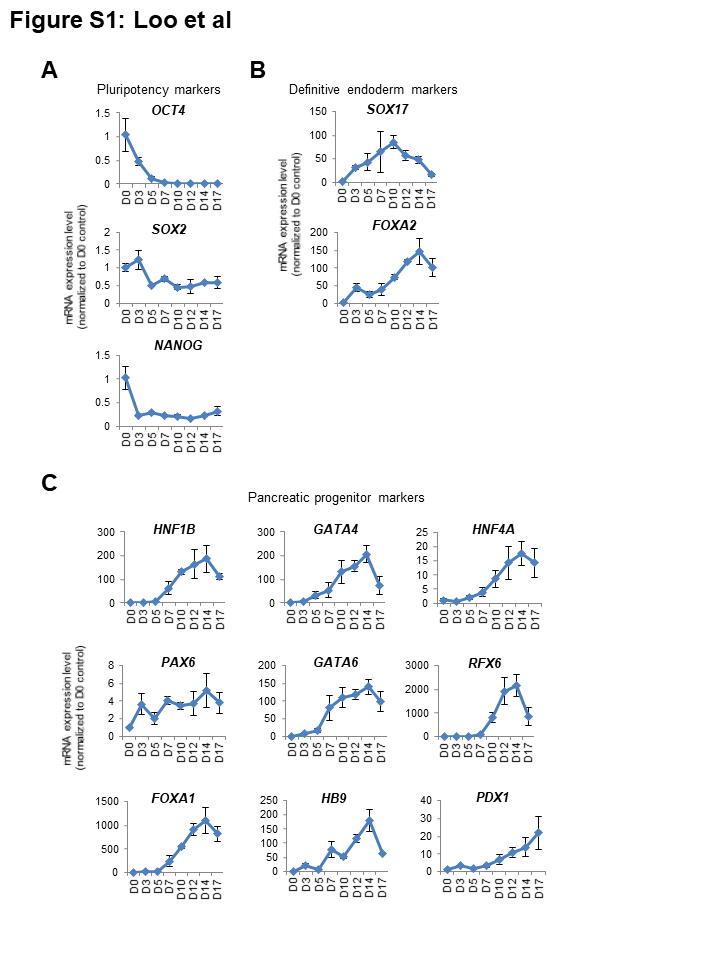

Supplement: Supplementary file 1 — Supplemental Figure 1 [file 41419_2020_2589_MOESM1_ESM.tif]

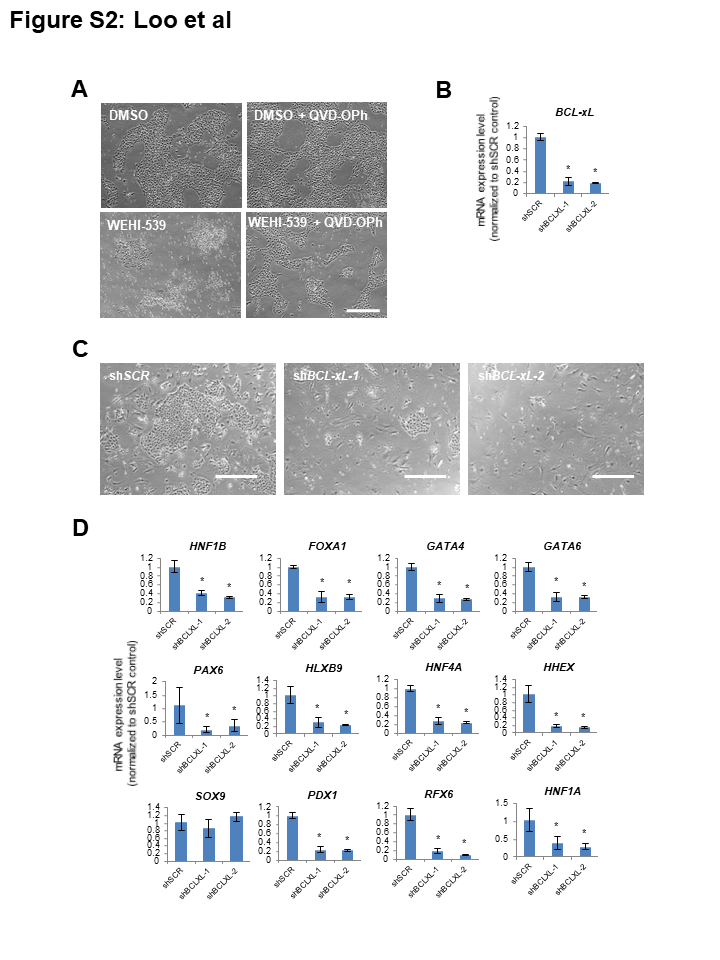

Supplement: Supplementary file 2 — Supplemental Figure 2 [file 41419_2020_2589_MOESM2_ESM.tif]

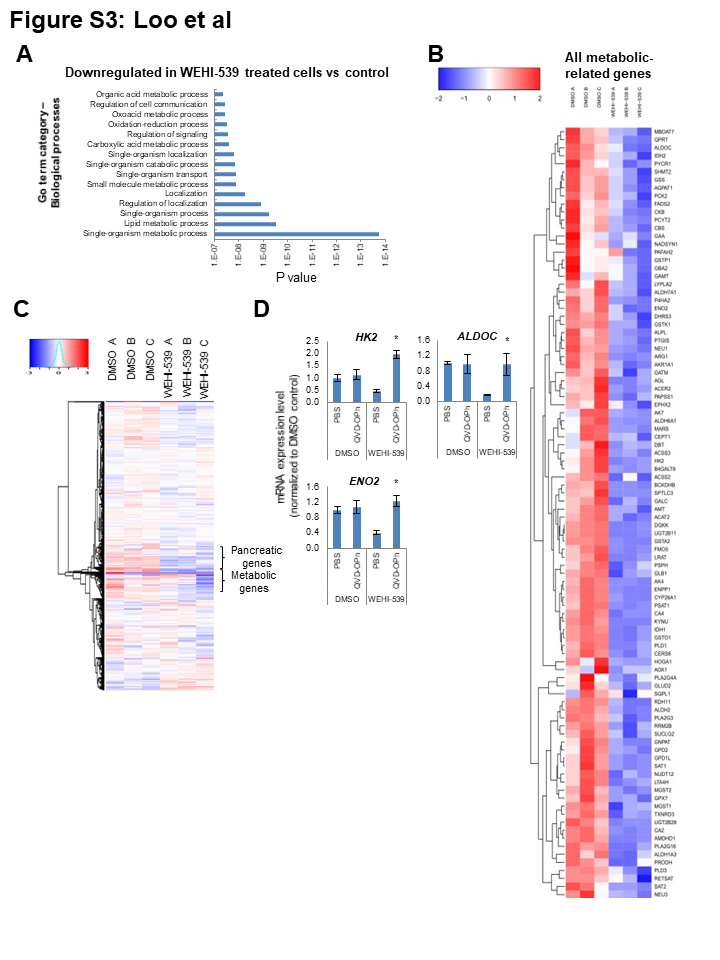

Supplement: Supplementary file 3 — Supplemental Figure 3 [file 41419_2020_2589_MOESM3_ESM.tif]

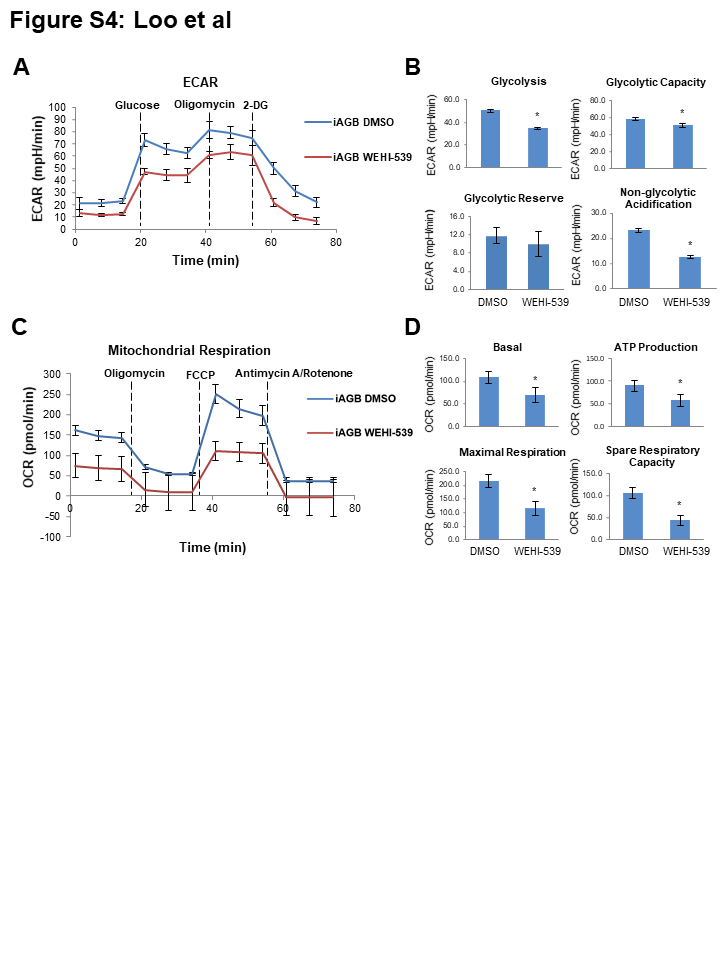

Supplement: Supplementary file 4 — Supplemental Figure 4 [file 41419_2020_2589_MOESM4_ESM.tif]

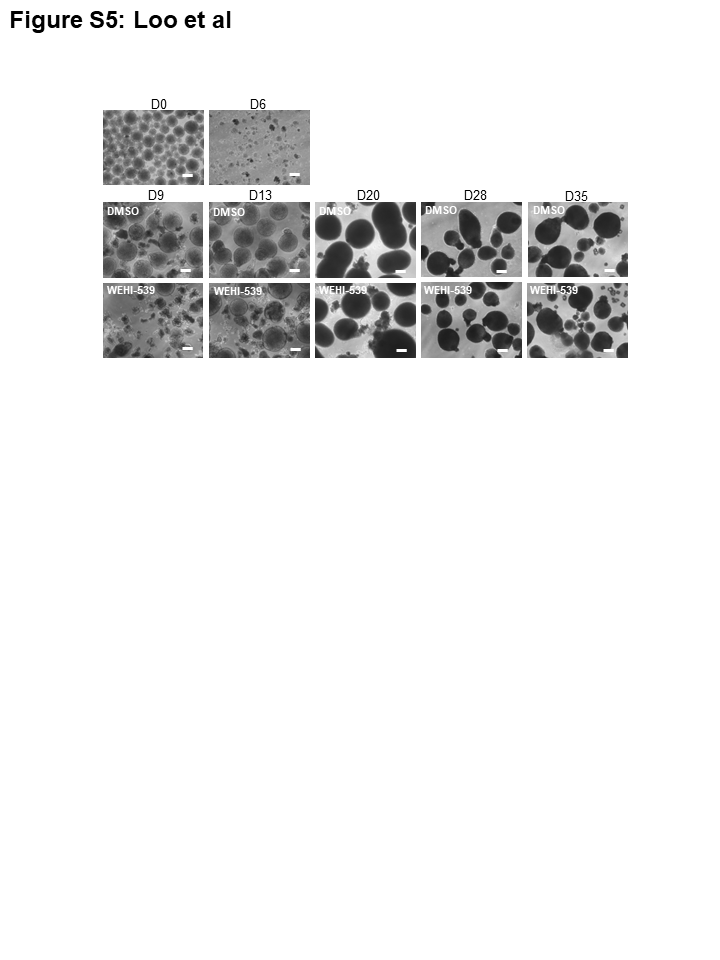

Supplement: Supplementary file 5 — Supplemental Figure 5 [file 41419_2020_2589_MOESM5_ESM.tif]
