# Supplementary material for: BCL-xL/BCL2L1 is a critical anti-apoptotic protein that promotes the survival of differentiating pancreatic cells from human pluripotent stem cells
Source: Cell Death Dis. 2020 May 18;11(5):378. doi: 10.1038/s41419-020-2589-7 (PMC7235254; doi:10.1038/s41419-020-2589-7)
Supplement: Supplementary file 7 — Supplemental Information- clean [file 41419_2020_2589_MOESM7_ESM.docx]

**SUPPLEMENTAL INFORMATION**

# **SUPPLEMENTAL FIGURE LEGENDS**

**Figure S1 (Related to Figure 1). Transcriptional profile of genes during 17D differentiation of hESCs.** Expression of (A) pluripotency, (B) definitive endoderm or (C) pancreatic gene transcripts over the course of 17D differentiation in H9 hESCs. Error bars indicate standard deviation of three biological replicates undergoing independent differentiations. A representative of at least two independent experiments is shown.

**Figure S2 (Related to Figures 2 and 3). Knockdown of *BCL2L1* decreased the expression of pancreatic genes.** (A) Brightfield images of D7 cells treated with DMSO/WEHI-539 in addition to QVD-OPh. Scale bar represents 500 μm. (B) Expression of *BCL2L1* transcripts upon knockdown with sh*BCL-xL*-1 or sh*BCL-xL*-2 on D7 cells. (C) Brightfield image of D10 cells knocked down for *BCL2L1* transcripts. Scale bar represents 200 μm. (D) Expression of pancreatic gene transcripts upon knockdown with sh*BCL-xL*-1 or sh*BCL-xL*-2 on D7 cells. Error bars indicate standard deviation of three biological replicates undergoing independent differentiations. Asterisk (*) indicates P < 0.05 compared to sh*SCR* control by two-way ANOVA. A representative of at least two independent experiments is shown.

**Figure S3 (Related to Figures 3, 4 and 5). RNA-Seq analyses revealed that the inhibition of BCL-xL function decreases the expression of pancreatic and metabolic genes.** (A) Gene ontology biological process for cells treated with WEHI-539 (downregulated). FC > 1.5; P < 0.05. (B) Global heatmap analysis of genes in D7 cells treated with DMSO or WEHI-539. (C) Hierarchical clustering heatmap analysis of metabolic genes in D7 cells treated with DMSO or WEHI-539. FC > 1.5; P < 0.05. Colors in the heat map depict gene expression in units of SD from the mean across all samples (upregulation in red, downregulation in blue). (D) Expression of metabolic gene transcripts upon treatment with WEHI-539 and QVD-OPh on D7 cells.

**Figure S4 (Related to Figure 5). BCL-xL function contributed to metabolic processes that occur during pancreatic specification.** (A) Glycolysis stress test and (B) individual component graphs of glycolysis, glycolytic capacity, glycolytic reserve and non-glycolytic acidification in D7 iAGb cells treated with DMSO or WEHI-539. (C) Mitochondrial respiration and (D) individual component graphs of basal mitochondrial respiration, ATP production, maximal respiration and spare respiratory capacity in D7 iAGb cells treated with DMSO or WEHI-539. Error bars indicate standard deviation of eight replicates. Asterisk (*) indicates P < 0.05 compared to DMSO control. A representative of at least two independent experiments is shown.

**Figure S5 (Related to Figure 6).** **Perturbation of BCL-xL early on during pancreas specification has detrimental long-term impact on pancreatic beta cell formation.** Brightfield image of cells treated with DMSO or WEHI-539 during 35D differentiation. Scale bar represents 200 μm.

# **SUPPLEMENTAL TABLE LEGENDS**

**Table S1: RNA-Seq analyses on DMSO and WEHI-539-treated D7 cells.**

**Table S2: Oligonucleotides utilized in this study.**

| **Gene** | **Usage** | **Accession Number** | **Forward Primer (5' to 3')** |
| --- | --- | --- | --- |
| *ALDOC* | QPCR | [NM_005165.2](https://www.ncbi.nlm.nih.gov/nuccore/NM_005165.2) | FW 5’ ACCCGAGCTGTGCTTGTGGC 3’ |
|  |  |  | RV 5’ TTGGCCATGCTGCCTACAGACTC 3’ |
| *BAD* | QPCR | NM_004322.3 | FW 5’ CGAGATCGGGCTTGGGGTGAG 3’ |
|  |  |  | RV 5’ CTGGGCCCTCATCTGTCTGCC 3’ |
| *BAK* | QPCR | NM_001188.4 | FW 5’ GATCCCGGCAGGCTGATCCC 3’ |
|  |  |  | RV 5’ TCCTGTTCCTGCTGATGGCGG 3’ |
| *BAX* | QPCR | NM_001291428.1 | FW 5’ GCCGGGTTGTCGCCCTTTTC 3’ |
|  |  |  | RV 5’ GCAGCCCCCAACCACCCTG 3’ |
| *BCL-xL* | QPCR | NM_001317919.1 | FW 5’ GGAGAACGGCGGCTGGGATA 3’ |
|  |  |  | RV 5’ GGCCACAGTCATGCCCGTCA 3’ |
| *BCL2* | QPCR | NM_000633.2 | FW 5’ AGGCTGGGATGCCTTTGTGGAA 3’ |
|  |  |  | RV 5’ CAAGCTCCCACCAGGGCCAAA 3’ |
| *BID* | QPCR | NM_197966.2 | FW 5’ GGGTAGTCGACCGTGTCCGC 3’ |
|  |  |  | RV 5’ GCTGGAACCGTTGTTGACCTCAC 3’ |
| *BIM* | QPCR | NM_138621.5 | FW 5’ GCAATGGCTTCCATGAGGCAGG 3’ |
|  |  |  | RV 5’ GTGGGTGGTCTTCGGCTGCT 3’ |
| β*-ACTIN* | QPCR | NM_001101.5 | FW 5’ TTGCCGATCCGCCGCCCGTC 3’ |
|  |  |  | RV 5’ CCCATGCCCACCATCACGCCCTGG 3’ |
| *BAD* | QPCR | NM_004322.3 | FW 5’ CGAGATCGGGCTTGGGGTGAG 3’ |
|  |  |  | RV 5’ CTGGGCCCTCATCTGTCTGCC 3’ |
| *ENO2* | QPCR | NM_001975.3 | FW 5’ CATTGCTCAGCTGGCCGGGA 3’ |
|  |  |  | RV 5’ GCTTGCACGCTTGGATGGCTT 3’ |
| *FOXA1* | QPCR | [NM_004496.4](https://www.ncbi.nlm.nih.gov/entrez/viewer.fcgi?db=nucleotide&id=1519243098) | FW 5’ AGCTACTACGCAGACACGCAGG 3’ |
|  |  |  | RV 5’ TGTTGCCGCTCGTAGTCATGGT 3’ |
| *FOXA2* | QPCR | NM_021784.4 | FW 5’ GGGCACGAGCCGTCCGACTGGA |
|  |  |  | RV 5’ GTTGCCCGAGCCGCTGCCCA 3’ |
| *GATA4* | QPCR | [NM_001308093.1](https://www.ncbi.nlm.nih.gov/entrez/viewer.fcgi?db=nucleotide&id=815890848) | FW 5’ GCAGAGAGTGTGTCAACTGTGGGG 3’ |
|  |  |  | RV 5’ TGGGGACCCCGTGGAGCTT 3’ |
| *GATA6* | QPCR | NM_005257.5 | FW 5’ GCCCCTCATCAAGCCGCAGA 3’ |
|  |  |  | RV 5’ CAAGTGGTCTGGGCACCCCAT 3’ |
| *HHEX* | QPCR | NM_002729.5 | FW 5’ ACACGCACGCCCTGCTCCGC 3’ |
|  |  |  | RV 5’ TGGCCAGACGCTTCCTCTCGGGC 3’ |
| *HK2* | QPCR | [NM_000189.4](https://www.ncbi.nlm.nih.gov/nuccore/NM_000189.4) | FW 5’ GGCACCCAGCTGTTTGACCAC 3’ |
|  |  |  | RV 5’ AGCCACAACGTCTCTGCCTTCC 3’ |
| *HLXB9* | QPCR | NM_005515.4 | FW 5’ GCGTCCACCGCGGGCATGATCC 3’ |
|  |  |  | RV 5’ AAGCGCTTGGGCCGCGACAGG 3’ |
| *HNF1A* | QPCR | [NM_001306179.1](https://www.ncbi.nlm.nih.gov/entrez/viewer.fcgi?db=nucleotide&id=807201166) | FW 5’ CTTCTGCAGGAGGACCCGTGGCGT 3’ |
|  |  |  | RV 5’ GGCGGCCCGCTTCTGCGTCT 3’ |
| *HNF1B* | QPCR | NM_000458.4 | FW 5’ GGGGCCCGCGTCCCAGCAAA 3’ |
|  |  |  | RV 5’ GGCCGTGGGCTTTGGAGGGGG 3’ |
| *HNF4A* | QPCR | NM_178849.2 | FW 5’ GGACGACCAGGTGGCCCTGCTCAGA 3’ |
|  |  |  | RV 5’ GCTCCGGGCAGTGCCGAGGGA 3’ |
| *MCL1* | QPCR | NM_021960.5 | FW 5’ AGGGCGACTTTTGGCCACCG 3’ |
|  |  |  | RV 5’ TGCCTTGGAAGGCCGTCTCG 3’ |
| *NANOG* | QPCR | NM_024865.4 | FW 5’ GACCTGGTGCACCCAATCCT 3’ |
|  |  |  | RV 5’ TCCAAGGCAGCCTCCAAGTC 3’ |
| *NOXA* | QPCR | NM_021127.2 | FW 5’ CCAGCAGAGCTGGAAGTCGAGTG 3’ |
|  |  |  | RV 5’ TGCAGTCAGGTTCCTGAGCAGAAG 3’ |
| *OCT4* | QPCR | NM_002701.6 | FW 5’ CCCCGGAGCCCTGCACCGTCA 3’ |
|  |  |  | RV 5’ CCCCCAGGGTGAGCCCCACATCG 3’ |
| *PAX6* | QPCR | NM_000280.4 | FW 5’ CCCACCACACCGGTTTCCTCC 3’ |
|  |  |  | RV 5’ GGTGGGCAGCATGCAGGAGT 3’ |
| *PDX1* | QPCR | NM_000209.4 | FW 5’ CCTTCCCGGAGGGAGCCGAGCC 3’ |
|  |  |  | RV 5’ GTAGGCCGTGCGCGTCCGCT 3’ |
| *PUMA* | QPCR | NM_001127240.2 | FW 5’ CCAGATTTGTGGTCCTCAGCCCT 3’ |
|  |  |  | RV 5’ TTGAGGTCGTCCGCCATCCG 3’ |
| *RFX6* | QPCR | NM_173560.4 | FW 5’ GCGGCTTGGAACAAGAGGCCA 3’ |
|  |  |  | RV 5‘ACGAGTGAAGCCACCCTCATTCTTT 3’ |
| *SFRP1* | QPCR | NM_003012.5 | FW 5’ GACCGGCCCATCTACCCGTG 3’ |
|  |  |  | RV 5’ CACACCGTTGTGCCTTGGGG 3’ |
| *SFRP2* | QPCR | NM_003013.3 | FW 5’ CAGCCACCGAGGAAGCTCCA 3’ |
|  |  |  | RV 5’ TCGGACACACCGTTCAGCTTGT 3’ |
| *SFRP4* | QPCR | NM_003014.4 | FW 5’ GCCATCGTCACGGACCTCCC 3’ |
|  |  |  | RV 5’ CACCGATCGGGGCTTAGGCG 3’ |
| *SFRP5* | QPCR | NM_003015.3 | FW 5’ CGCCTCCAGTGACCAAGATCTGC 3’ |
|  |  |  | RV 5’ GTGTCCTTGCGCTTCAGGGGG 3’ |
| *SOX2* | QPCR | NM_003106.4 | FW 5’ GACGGAGCTGAAGCCGCCGGG 3’ |
|  |  |  | RV 5’ CGCTGCCCGCGGGACCACAC 3’ |
| *SOX9* | QPCR | NM_000346.4 | FW 5’ ACCAGCCGCGGCGGAGGAAGT 3’ |
|  |  |  | RV 5’ GGGATTGCCCCGAGTGCTCGCC 3’ |
| *SOX17* | QPCR | NM_022454.4 | FW 5’ GGCGAGGCGCCGGCGAACAG 3’ |
|  |  |  | RV 5’ TCAGCGCCTTCCACGACTTGCCCAG 3’ |
| *WNT2B* | QPCR | NM_024494.2 | FW 5’ ATGCTGAGACCGGGTGGTGC 3’ |
|  |  |  | RV 5’ AATGTACCACCAGGACGTGTCTACG 3’ |
| *WNT5A* | QPCR | [NM_003392.4](https://www.ncbi.nlm.nih.gov/nuccore/NM_003392.4) | FW 5’ GCTCGCTCGGGTGGCGA 3’ |
|  |  |  | RV 5’ CCTAGCGACCACCAAGAATTGGCT 3’ |
| *WNT5B* | QPCR | [NM_032642.2](https://www.ncbi.nlm.nih.gov/nuccore/NM_032642.2) | FW 5’ CTGGAGCCTGATGGACGGGTG 3’ |
|  |  |  | RV 5’ AGCTAATGACCACCAGGAGTTGGC 3’ |
| *WNT7B* | QPCR | [NM_058238.2](https://www.ncbi.nlm.nih.gov/nuccore/NM_058238.2) | FW 5’ ACGTGAAGCTCGGAGCACTGT 3’ |
|  |  |  | RV 5’ AGCGTCCGAAGCGGAACTGG 3’ |
| *WNT8B* | QPCR | [NM_003393.3](https://www.ncbi.nlm.nih.gov/nuccore/NM_003393.3) | FW 5’ GCTTCGCAGTGCCAATCGGG 3’ |
|  |  |  | RV 5’ TTGCCCGTTGCGGGAGTCAT 3’ |
| sh*BCL-xL* 1^st^ pair | RNAi | N/A | CCGGGTGGAACTCTATGGGAACAATCTCGAGATTGTTCCCATAGAGTTCCACTTTTTG |
|  |  |  | AATTCAAAAAGTGGAACTCTATGGGAACAATCTCGAGATTGTTCCCATAGAGTTCCAC |
| sh*BCL-xL* 2^nd^ pair | RNAi | N/A | CCGGGCTCACTCTTCAGTCGGAAATCTCGAGATTTCCGACTGAAGAGTGAGCTTTTTG |
|  |  |  | AATTCAAAAAGCTCACTCTTCAGTCGGAAATCTCGAGATTTCCGACTGAAGAGTGAGC |
